# Comparative Study of Classification Algorithms for Various DNA Microarray Data

**DOI:** 10.3390/genes13030494

**Published:** 2022-03-11

**Authors:** Jingeun Kim, Yourim Yoon, Hye-Jin Park, Yong-Hyuk Kim

**Affiliations:** 1 Department of IT Convergence Engineering, Gachon University, Seongnam-daero 1342, Seongnam-si 13120, Korea; wlsrms27@gachon.ac.kr; 2 Department of Computer Engineering, College of Information Technology, Gachon University, Seongnam-daero 1342, Sujeong-gu, Seongnam-si 13120, Korea; 3 Department of Food Science and Biotechnology, College of BioNano Technology, Gachon University, Seongnam-daero 1342, Sujeong-gu, Seongnam-si 13120, Korea; nimpi79@hanmail.net; 4 School of Software, Kwangwoon University, 20 Kwangwoon-ro, Nowon-gu, Seoul 01897, Korea; yhdfly@kw.ac.kr; 5 Department of Cell and Regenerative Biology, School of Medicine and Public Health, University of Wisconsin-Madison, 1111 Highland Ave, Madison, WI 53705, USA

**Keywords:** classification, microarray, machine learning, multilayer perceptron, random forest, decision tree, support vector machine, *k*-nearest neighbors

## Abstract

Microarrays are applications of electrical engineering and technology in biology that allow simultaneous measurement of expression of numerous genes, and they can be used to analyze specific diseases. This study undertakes classification analyses of various microarrays to compare the performances of classification algorithms over different data traits. The datasets were classified into test and control groups based on five utilized machine learning methods, including MultiLayer Perceptron (MLP), Support Vector Machine (SVM), Decision Tree (DT), Random Forest (RF), and *k*-Nearest Neighbors (KNN), and the resulting accuracies were compared. *k*-fold cross-validation was used in evaluating the performance and the result was analyzed by comparing the performances of the five machine learning methods. Through the experiments, it was observed that the two tree-based methods, DT and RF, showed similar trends in results and the remaining three methods, MLP, SVM, and DT, showed similar trends. DT and RF generally showed worse performance than other methods except for one dataset. This suggests that, for the effective classification of microarray data, selecting a classification algorithm that is suitable for data traits is crucial to ensure optimum performance.

## 1. Introduction

Microarrays have been developed by combining modern mechanical and electrical engineering technologies with the existing knowledge in molecular biology. While the traditional methods allowed researchers to measure the expression of a small number of genes at a time, the introduction of microarrays enabled the expression analysis of tens of thousands of genes in a single experiment. This led to the development of experimental techniques that were capable of generating a large volume of genomic information from a single cell [1]. Since various genes in an organism mutually affect and regulate their expressions, microarray data can be used as a tool to analyze specific diseases.

Microarray-based analysis methods can be broadly classified into five categories: analysis of differentially expressed genes, analysis of differentially expressed gene pairs, functional annotation, clustering analysis, and classification analysis [2,3,4,5]. In this study, classification analysis was selected for evaluation. The classification analysis method is a widely used multivariate statistical method that can be used to determine or predict classes of unknown groups of data. This method has typically been used to analyze cancer microarray data, and many recent studies have accurately classified acute myeloid leukemia and acute lymphoblastic leukemia using this method [6].

Meanwhile, in recent years, classification analysis using RNA-seq data has frequently been conducted [7,8,9,10]. RNA-Seq has advantages for examining transcriptome fine structure and does not depend on genome annotation for prior probe selection so that it can avoid the related biases [11,12]. However, it also has some disadvantages. Protocols for RNA-Seq are still not fully optimized and it requires high cost and high-power computing facilities. Additionally, if paralogues are present, analysis of the data can be complex. On the contrary, microarray has well-defined protocols and is relatively low in cost. Standardized approaches for data are possible with microarray [13]. Therefore, microarray-related studies are still underway [14,15,16,17,18].

The microarray data used in this study included datasets of samples categorized on the following bases. First, the datasets representing the presence or absence of a specific disease were included. Second, the datasets containing either of two similar diseases were selected for this study. Third, the datasets containing samples obtained from people with occupations entailing exposure to harmful environments, such as fine dust, and those obtained from people with other occupations, which are for the control group, were included. Furthermore, two types of data, i.e., miRNA data and RNA data, were analyzed. The application of machine learning algorithms to this variety of microarray datasets can provide a better understanding of the performance of machine learning with microarray data. The machine learning algorithms used for classifying microarray data were implemented in Python, followed by cross-validation to calculate the accuracy as a metric of algorithmic performance.

The rest of the paper is organized as follows: Section 2 describes data sets, data preprocessing, and classification algorithms; Section 3 shows the results of the classification algorithm for each data; Section 4 summarizes and discusses the classification results; and, finally, Section 5 concludes this work.

## 2. Materials and Methods

### 2.1. Data Acquisition

Microarrays are highly dense arrays of DNA molecules with known nucleotide sequences aggregated on a small slide [19]. Microarrays may be used to assess the overall expression of a large number of genes and contribute to a genome-based analysis of biological phenomena [20,21]. MicroRNAs (miRNAs) are short RNA molecules consisting of approximately 22 nucleotides and are involved in the post-transcriptional regulation of gene expression. miRNAs are known to regulate the expression of over 60% of human genes and are associated with various diseases [22].

The miRNA and RNA datasets were acquired from the Gene Expression Omnibus repository at the National Center for Biotechnology Information (GEO-NCBI) [23]. Among the datasets available for lung cancer, chronic obstructive pulmonary disease (COPD), and cardiovascular disease (CVD), wherein each representative disease was induced by exposure to fine dust, we acquired four datasets with clear control groups and relatively large sample sizes. Furthermore, we acquired two microarray datasets that compared the profiles of occupations with and without exposure to fine dust, respectively. Some of the datasets used in this study also included information on smoking, sex, age, and BMI, in addition to miRNA and RNA. While all datasets were associated with exposure to a fine dust to some extent, their classification criteria and data traits were distinct as follows:

Dataset 01, titled “MicroRNA profiling of chronic lung disease for the Lung Genomics Research Consortium”, provided miRNA data on patients with COPD and interstitial lung disease (ILD), which are two similar pulmonary diseases. COPD is a lung disease caused by repeated exposure to a noxious agent resulting in irreversible airflow limitation and ILD is a loosely defined group of diseases characterized by changes in the interstitium of the lung, causing pulmonary restriction and impaired gas exchange [24].

Dataset 02, titled “The site and nature of terminal bronchiolar destruction in chronic obstructive pulmonary disease (COPD)”, provided RNA data of patients with COPD besides that of healthy subjects [25]. 

Dataset 03, titled “Genome-wide screening of transcriptional modulation in non-smoking female lung cancer in Taiwan”, provided RNA data of non-smoking females with lung cancer and those without lung cancer [26]. 

Dataset 04 was titled “Differences in monocyte miRNA profiles between patients with coronary artery disease and healthy controls”. This dataset included miRNA data from healthy subjects and patients with CVD, which is one of the primary causes of death in humans [27]. 

Dataset 05 was titled “Transcriptomic changes in the nasal epithelium associated with diesel engine exhaust exposure”. Diesel engine exhaust (DEE) is one of the primary causes of air pollution worldwide, which can affect the human body. This RNA dataset was collected from those working in diesel engine factories, along with those working without DEE exposure as the control group, for a detailed analysis of genes affected by air pollution [28]. 

Dataset 06, titled “Expression of viral and human microRNAs in blood in the Beijing Truck Driver Air Pollution Study”, provided miRNA data collected from the blood samples of truck drivers exposed to air pollution, with that of office workers as the control group. This dataset was constructed to investigate the association between air pollution exposure and clinical outcomes [29]. 

Since the sample traits listed in the six datasets not only included occupation but also BMIs and smoking status, we were able to conduct classification experiments on various features, such as truck driver/office worker, obese/non-obese, and smoking/non-smoking. All datasets used for the experiments are listed in Table 1 with their titles, classification groups, variable counts, and sample counts.

Table 2 shows an example microarray data (miRNA data indicating the effects of air pollution on truck drivers) used in the experiments. The rows specify the gene names and values, whereas the columns specify the sample names. The miRNA data used in the experiments represent the expression of each gene for each sample in a matrix form.

### 2.2. Data Preprocessing

Feature scaling is a kind of data preprocessing to improve the performance of machine learning algorithms [30]. Normalization and standardization are the two most commonly used feature scaling techniques in machine learning. Normalization rescales the values into a range of [0, 1] and standardization rescales data to have a mean of 0 and a standard deviation of 1. The choice of normalization or standardization depends on data and machine learning algorithms. There is no simple rule that determines when to use normalization or standardization. Therefore, it is necessary to apply both methods and compare the results to know which method is better [31]. In this study, only normalization was applied, because normalization showed generally good performance according to the comparison. However, there were cases where standardization was better than normalization, and the results according to these feature scaling methods were attached to Appendix A.

The equation for normalization used in this study is given as follows:

(1) $$x_{i}{}^{new}:=\frac{x_{i}-x_{min}}{x_{max}-x_{min}}$$

If there are no negative values in the dataset, the values are scaled to a range between 0 and 1, and if there are negative values, the values are scaled to a range between −1 and 1. As the microarray datasets used in this study did not contain any negative values, all of them were scaled to a range [0, 1].

### 2.3. Classification Algorithm

Machine learning is a subfield of artificial intelligence in which algorithms are developed to allow systems to train based on a given dataset and execute activities that are not specified in the code [32] Machine learning can be further categorized into supervised or unsupervised learning based on whether or not the given data are labeled. In supervised learning such as support vector machines, decision trees, and neural networks, systems use the features of the given data to predict their labels. On the other hand, in unsupervised learning such as clustering, the system is trained entirely on the unlabeled input values. In this study, supervised learning was used to classify the microarray data.

MLP is a layered neural network with one or more hidden layers between the input and output layers [33]. The network is a feedforward network in which the layers are directed as per the order: input, hidden, and output layers, and there is no connection between the nodes of the same layer or between the output and input layers [34]. MLP uses activation functions, typically the sigmoid or rectified linear unit (ReLU) functions. The sigmoid function provides a value between 0 and 1 as output, enabling the neural network to make subtle classifications of the data. However, this characteristic of the sigmoid function poses a disadvantage, i.e., with deeper networks; the output of the function is heavily biased towards either end of the range, leading to a derivative value close to 0. To solve this problem, the ReLU function was proposed, which returns 0 for an input value smaller than 0 but returns the original input value for one larger than 0. This does not lead to derivative values converging to zero, even with deeper networks. The equation for the ReLU function is as follows:

(2) $$f\left(x\right)=x^{+}\equiv \max\left(0,x\right)=\{\begin{array}{c} \;0\;\left(x<0\right)\\ \;x\;\left(x\geq 0\right) \end{array}$$

where x is the input to a neuron.

Some optimizers that enhance and stabilize the learning rates of MLP include stochastic gradient descent, momentum, nesterovated gradient, and adaptive moment estimation (Adam). Adam was selected for this study, owing to its high computational efficiency, low memory requirements, and scalability in large datasets [35]. The default value of the learning rate, which controls the step size in weight updates, was set to 0.01 since the learning rate of 0.01 is known to be effective in preventing underfitting [36].

SVM is a machine learning algorithm proposed by Vapnik [37]. It is a highly generalizable classifier typically used for classification and regression analysis. SVM classifies a set containing elements of various classes in an *N*-dimensional space into several subsets using the (*N*-1)-dimensional hyperplane of the maximum margin [38]. The algorithm is currently being used in a wide range of fields including text, handwriting, facial, object recognition, and bioinformatics [39]. The SVM provides different outputs based on the values of two parameters: *C*, specifying the degree of error permitted, and γ, specifying the curvature of the boundary.

In this study, the RBF (radial basis function)-SVM was used for its specialization in nonlinear factors. The RBF kernel function is expressed as follows [40]:

(3) $$K\left(x_{i},x_{j}\right)=\exp\left(-\gamma \|x_{i}-x_{j}\|\right){}^{2}$$

where $\|x_{i}-x_{j}\|{}^{2}$ is the squared Euclidean distance between the two vectors $x_{i}$ and $x_{j}$.

The RBF kernel requires predetermined values for the *C* and γ parameters since the *C* and γ value that shows the highest performance varies according to the size of the dataset. The value with the highest performance among 0.001, 0.01, 0.1, 1, 10, and 100 was selected to obtain experimental results. The six values are the most generally used for the γ value of RBF-SVM.

DT is an analytical method in which a tree-like structure consisting of decision rules is constructed to classify data into several subsets [41]. A tree is a collection of layered nodes and branches. Top-down algorithms are generally used to construct decision trees, and the selection of classification variables and threshold values is crucial in each step of constructing the tree from the top to the bottom. Without a limit on the depth of the decision tree, the tree can become infinitely deep and complex. Thus, trees without pruning may result in overfitting, which may be prevented by limiting the tree depth. In this study, the maximum tree depth parameter, max_depth, was set to 3 to avoid overfitting [42].

RF is an ensemble classification algorithm based on decision trees trained on randomly selected subsets of data and features. The number of trees in the forest was limited to 100 since a forest with more than 100 trees is known to be able to cause overfitting [43]. For each node, the random forest randomly selects explanatory variables and provides the optimal output using the set of selected explanatory variables. However, the algorithm is unstable due to the instability in datasets or variability in classifiers, which cause even a small change in data to lead to a different result. To circumvent this issue, RF is usually performed with bagging and bootstrapping [44].

KNN algorithm, proposed by Cover and Hart in 1968, is a nonparametric method used in classification or regression [45]. KNN intuitively classifies unlabeled samples based on the inter-sample similarity observed in the training set. A small value of the number of neighbors provided as a parameter leads to a complex decision boundary in the model and consequent overfitting, while a large value leads to a simple decision boundary and underfitting. Thus, it is important to determine an appropriate value for this parameter. In this study, the value showing the highest performance was set as the value of the core parameter of KNN, n_neighbors, individually for each dataset.

Cross-validation refers to averaging the performances of *k* models, each generated from a different partition of the dataset. *k*-fold refers to partitioning the dataset into *k* subsets using *k*-1 of them as the training sets and the remaining as the validation set. This process is repeated *k* times. The evaluation of models using this approach ensures that the entire data is used as both training and validation data, leading to a lower likelihood of overfitting. As usual, we set *k* as 10, partitioning the dataset into 10 subsets, for performing cross-validation.

In this study, the accuracy of the classification model is defined as

(4) $$Accuracy=\frac{1}{n}\overset{n-1}{\underset{0}{\sum }}1({\hat{y}}_{i}=y_{i})$$

where n is the number of samples, ${\hat{y}}_{i}$ is the predicted value of the i-th sample, $y_{i}$ is the corresponding actual value, and 1(x) is an indicator function.

Table 3 shows the types of classification algorithms used in the experiment, parameters used in algorithm design, and values used for parameters. We conducted nested cross-validation for parameter tuning and evaluation of SVM and KNN. For each fold of cross-validation, these two classification algorithms were tested with different parameter values to achieve optimal performance. For the other three classification algorithms, MLP, DT, and RF, predetermined values known to be effective in improving performance were used for parameter values [36,42,43]. For the SVM model, a model with a linear SVM kernel was used, and the C and γ values were obtained through a grid search of the training set in each fold resulting in different values across models. For the KNN model, a value between 1 and 58 was specified for each model as the number of neighbors used to find the optimal value. Thus, the range of the parameter values and the optimal k value were different for each model. The MLP classification model generated two hidden layers since it has been verified effective in other studies for disease diagnosis [46]. Each hidden layer contained 10 neurons and used the ReLU activation function. Adam was used as the gradient descent algorithm with an initial learning rate of 0.01 and was executed over 500 epochs. The DT model was generated with a maximum tree depth of three. The RF model generated a random forest of 100 trees.

Models using MLP, SVM, DT, RF, and KNN were implemented in Python, as mentioned earlier, and the source code was attached as File S1. Optimal models were developed by tuning the parameters. All possible parameter values were tested, especially for SVM and KNN, and the results were compared to each other. The classification models were trained and tested using the system with NVIDIA Tesla K80 GPU, Intel Core i5-6200 CPU @ 2.30 GHz, and 8 GB memory.

## 3. Results

### 3.1. Classification of Lung Disease Data (COPD/ILD)

Dataset 01 provided miRNA data for patients with COPD or ILD, which are two similar pulmonary diseases. All samples of the patients diagnosed with ILD or COPD were obtained from the Lung Tissue Research Consortium (LRTC). The dataset included 319 subjects, of which 183 had ILD and 136 had COPD [24,47].

**Figure 1:**
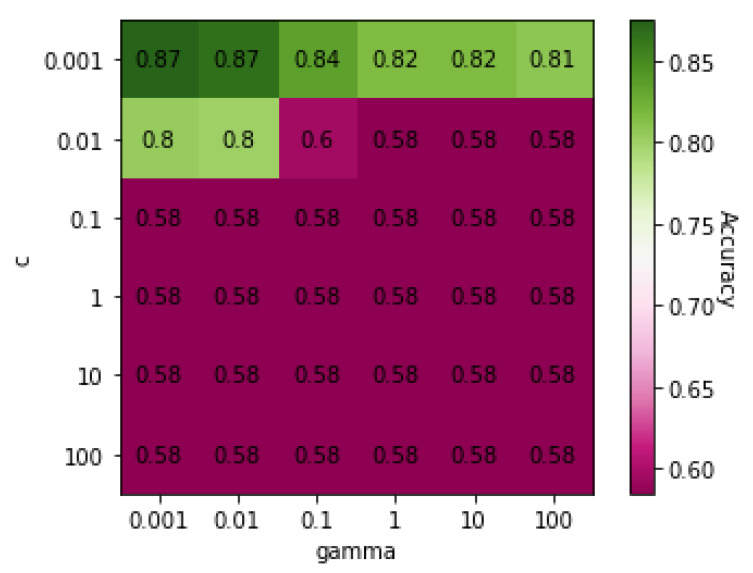
Heatmap of values of SVM parameters *C* and γ on Dataset 01.

Table 4 shows the classification results for COPD and ILD obtained using five different algorithms: MLP, RF, DT, SVM, and KNN. The results show that SVM, MLP, and KNN had accuracies of over 80%. Figure 1 shows a heatmap of SVM accuracies with different values of *C* and γ, the two core parameters of SVM, displayed on a color gradient. SVM achieved the highest accuracy when both the *C* and γ values were 0.001.

RF and DT, using tree structures, showed lower accuracy than the others. RF had a lower accuracy (73.3%), and all five classification algorithms classified the patients with COPD from those with ILD with more than 70% accuracy.

### 3.2. Classification of COPD Data (COPD/Control)

Dataset 02 included microarray data from 77 patients with COPD and 40 healthy subjects [25].

**Figure 2:**
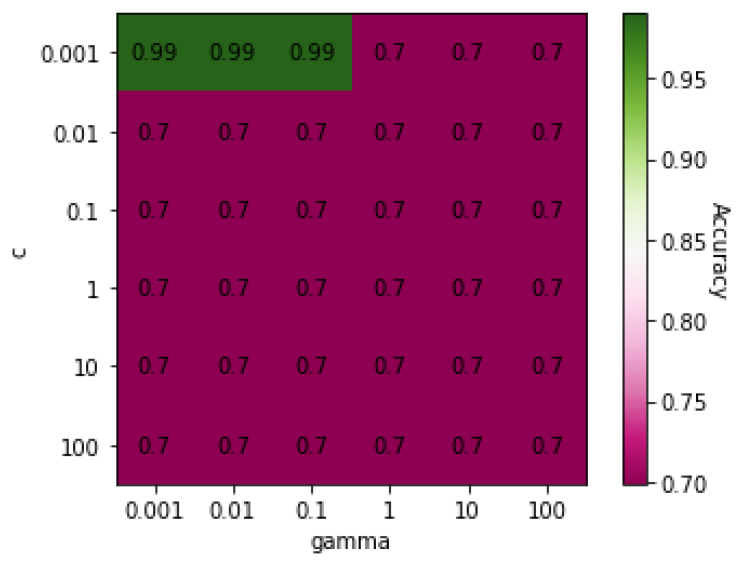
Heatmap of values of SVM parameters *C* and γ on Dataset 02.

Table 5 shows the classification results for COPD and ILD using five different algorithms: MLP, RF, DT, SVM, and KNN. SVM had the highest accuracy (99%) in classifying the subjects based on the presence or absence of COPD. The heatmap (Figure 2) shows that the highest accuracy was achieved with a *C* value of 0.001 and a γ value of 0.001, 0.01, or 0.1.

While RF had the lowest accuracy (68.3%), the algorithms generally had accuracies over 80%, thus classifying the subjects with a disease satisfactorily.

### 3.3. Classification of Lung Cancer Data (Lung Cancer/Control)

Dataset 03 provided miRNA data for healthy subjects and patients with lung cancer [26]. This dataset was used to comparatively analyze non-smoking female patients with lung cancer and healthy subjects [48,49].

Table 6 shows the classification results between patients with lung cancer and healthy subjects using the five classification algorithms. The DT and SVM showed high accuracies of 95% each. The lowest accuracy was 73%, suggesting that all algorithms classified between the two groups with an accuracy of more than 73%.

**Figure 3:**
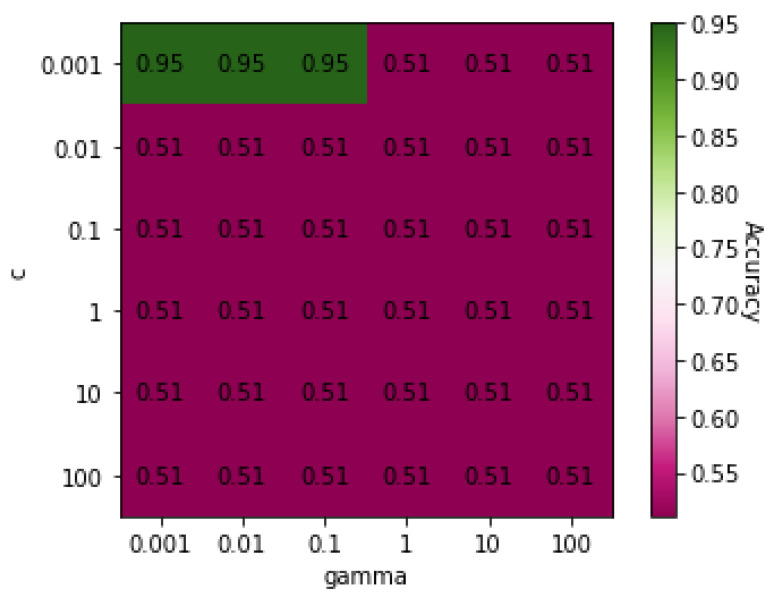
Heatmap of values of SVM parameters *C* and γ on Dataset 03.

Figure 3 shows a heatmap of the SVM accuracies with different *C* and γ values displayed on a color scale. The highest accuracy was achieved with a *C* value of 0.001 and γ values of 0.001, 0.01, or 0.1. The lowest accuracy was 73%, suggesting that all algorithms classified between the two groups well.

### 3.4. Classification of CVD Data (CVD/Control)

Dataset 04 provided the miRNA data of 40 males with premature CVD and 40 healthy males of the same age, measured using microarrays [27,50,51]

Table 7 shows the classification results between the CVD and control using the five classification algorithms. All algorithms were able to classify between the patients with CVD and healthy subjects with an accuracy of over 50%. However, SVM had the highest accuracy (77%), which was achieved with a *C* value of 0.001 and a γ value of 0.01.

**Figure 4:**
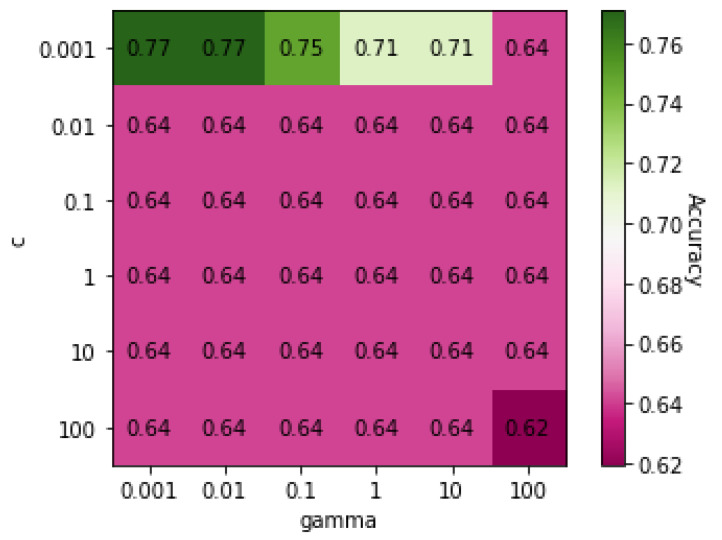
Heatmap of values of SVM parameters *C* and γ on Dataset 04.

Figure 4 shows a heatmap of the SVM accuracies with different *C* and γ values displayed on a color scale. The highest performance was observed when the *C* value was 0.001, and the γ value was 0.01.

### 3.5. Classification of Diesel-Exposure Data (Diesel Factory Worker/Control)

Dataset 05 provided RNA data of factory workers exposed to DEE and those not exposed to DEE [28,52]. The algorithms attempted to classify these using microarray data.

Table 8 shows the classification results of the diesel factory workers and control subjects using the five classification algorithms.

**Figure 5:**
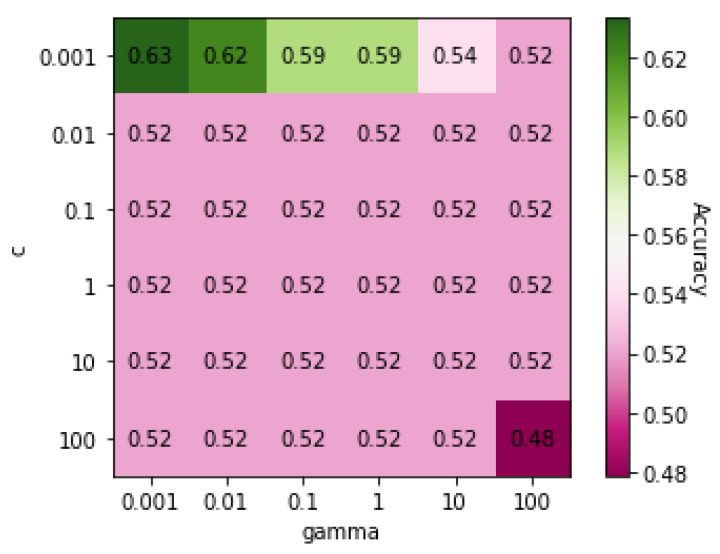
Heatmap of values of SVM parameters *C* and γ on Dataset 05.

**Figure 6:**
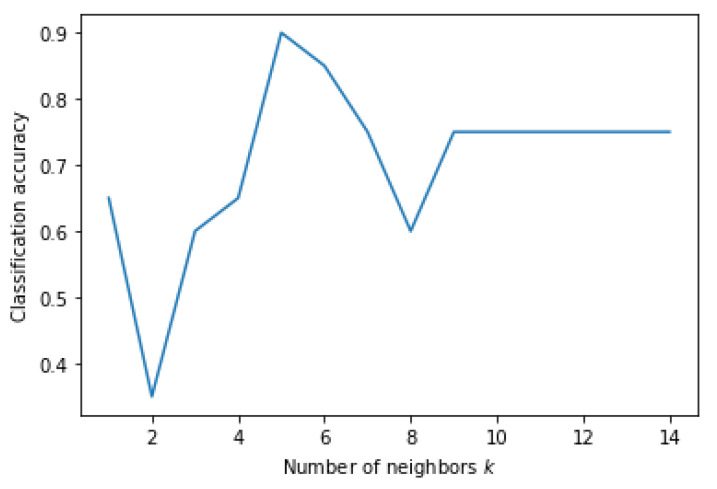
Classification accuracy according to *k* parameter of KNN on Dataset 05.

Figure 5 shows a heatmap of the SVM accuracies with different *C* and γ values displayed on a color scale. The highest performance was achieved when the *C* and γ values were both 0.001. Figure 6 shows the accuracy of the KNN classification algorithm obtained at different values of *k* plotted as a graph. KNN achieved the highest accuracy, with a *k* value of five. MLP and KNN showed high accuracies in this experiment. However, the tree-structure algorithms, random forest, and decision tree failed to classify this data well with an accuracy of less than 50%.

### 3.6. Classification of Occupation Data (Truck Driver/Office Worker)

Dataset 06 provided miRNA data on truck drivers with high exposure to air pollution, as well as office workers with relatively low exposure to air pollution [29]. All participants were residents of the Beijing metropolitan area and had been working for at least two years at the same location at the time of data collection. None of the participants took any regular medications, such as anti-inflammatory drugs or aspirin. For each participant, this dataset provided miRNA data collected over two days with an interval of 1–2 weeks in between [53,54].

This dataset listed occupations, BMIs, and information on whether the subjects smoked or not as traits of each sample. This enabled us to conduct further classification experiments, such as truck driver/office worker, obese/non-obese, and smoking/non-smoking. Using this dataset, we classified the differences between truck drivers and office workers.

**Figure 7:**
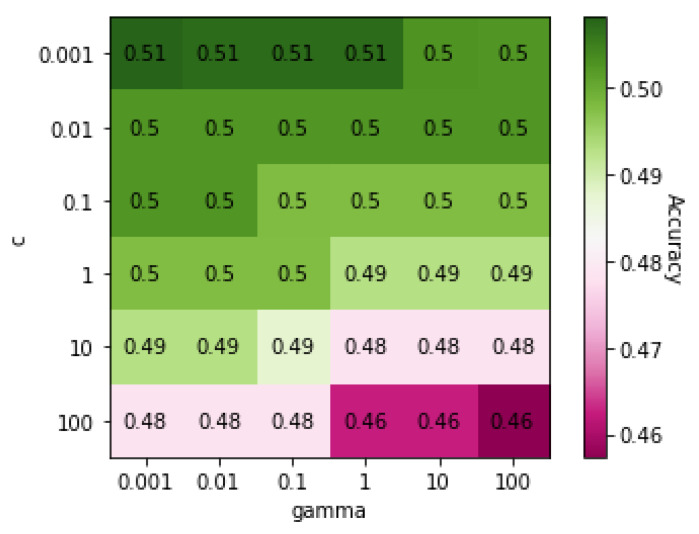
Heatmap of values of SVM parameters *C* and γ on Dataset 06 when classification was based on occupation.

Table 9 shows the classification results for truck drivers and office workers using the five classification algorithms. In general, the accuracies were low in classifying occupations. Figure 7 shows a heatmap of the SVM accuracies with different *C* and γ values displayed on a color scale. The highest accuracy was achieved when both *C* and γ values were 0.001.

**Figure 8:**
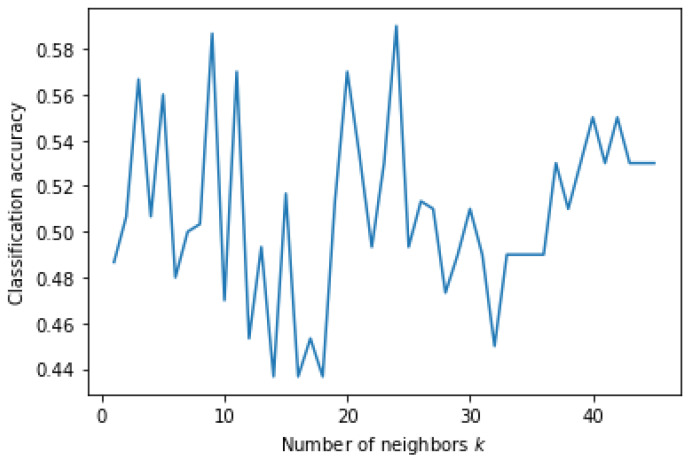
Classification accuracy according to *k* parameter of KNN on Dataset 06 when classification was based on occupation.

In this experiment, KNN showed the highest accuracy among the five classification algorithms. Figure 8 shows the accuracy of the KNN classification algorithm at different values of *k* plotted as a graph. KNN achieved the highest accuracy with a *k* value of 24. The decision tree correctly classified less than half of the dataset.

In the next experiment, the algorithms were applied to classify the same dataset by obesity (obese/non-obese). In this experiment, subjects with a BMI of 25 or higher were classified as obese. The WHO (World Health Organization, Geneva, Switzerland) uses the cutoff point of BMI for defining obesity as 30 [55], however, the lower cutoff point of 25 is usually used for identifying obesity for Asians [56].

Table 10 shows the classification results of the obese and control subjects using the five classification algorithms. The algorithms used miRNA data to classify obese and non-obese subjects better than when classifying the subjects by occupation.

**Figure 9:**
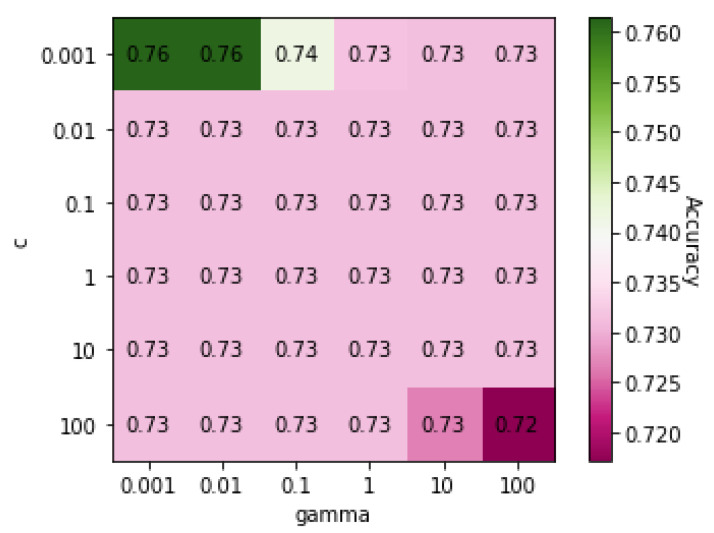
Heatmap of values of SVM parameters *C* and γ on Dataset 06 when classification was based on obesity.

**Figure 10:**
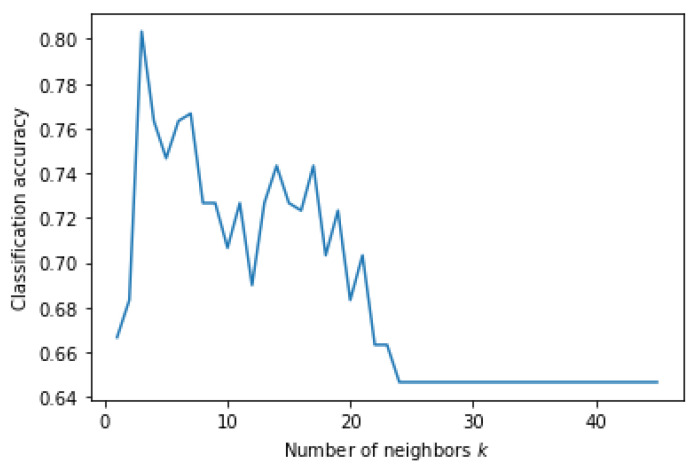
Classification accuracy according to *k* parameter of KNN on Dataset 06 when classification was based on obesity.

Figure 9 shows a heatmap of the SVM accuracies with different *C* and γ values displayed on a color scale. The highest accuracy was achieved with a *C* value of 0.001 and a γ value of 0.001 or 0.01. Figure 10 shows the accuracy of the KNN classification algorithm at different values of *k* plotted as a graph. The best performance was recorded with a *k* value of 3, and other algorithms also performed the classification adequately.

The next experiment also used the same dataset to classify the subjects based on whether or not they smoked.

Table 11 shows the classification results between smokers and non-smokers using the five classification algorithms. The classification results based on smoking showed lower accuracies than those obtained based on obesity but higher than those obtained based on occupation.

**Figure 11:**
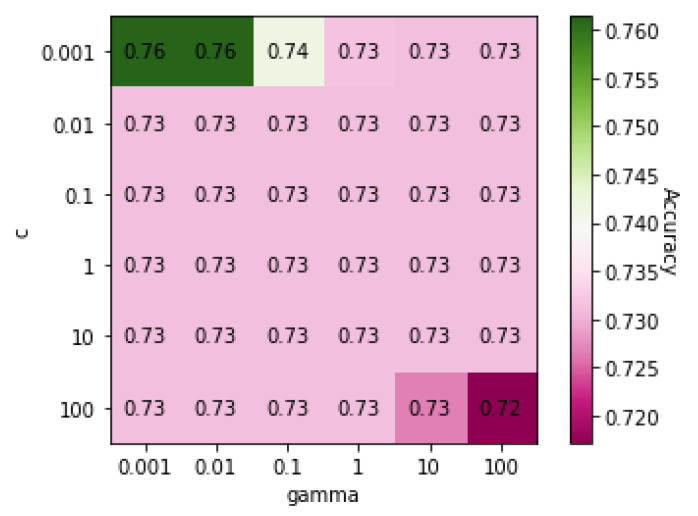
Heatmap of values of SVM parameters *C* and γ on Dataset 06 when classification was based on smoking habits.

**Figure 12:**
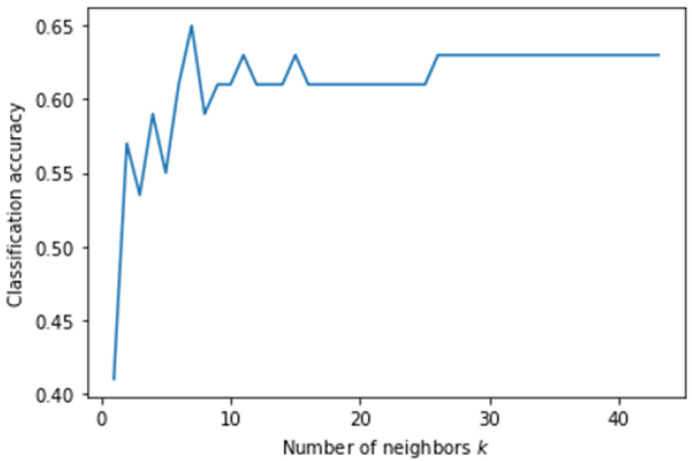
Classification accuracy according to *k* parameter of KNN on Dataset 06 when classification was based on smoking habits.

Figure 11 shows a heatmap of SVM accuracies with different *C* and γ values displayed on a color scale. The highest accuracy was achieved when the *C* and γ values were both 0.001. Figure 12 shows the accuracy of the KNN classification algorithm at different values of *k* represented as a graph. KNN and DT classified the data adequately, with KNN achieving the highest accuracy, with a *k* value of eight.

## 4. Discussion

Experiments were conducted to classify microarray data into two groups using machine learning. In the first experiment, wherein incidences of COPD and ILD were classified, all five algorithms distinguished two groups with an accuracy of more than 70%. The second experiment aimed to classify the data regarding the differences between patients with COPD and healthy subjects. In this experiment, SVM showed the highest accuracy (99%) in contrast to RF (random forest), which showed the lowest accuracy (68.3%). In the third experiment, classification was carried out to differentiate between lung cancer patients and healthy people, and the DT (decision tree) and SVM models showed high accuracies. In the fourth experiment, subjects in the dataset were classified based on the difference between patients with CVD and control groups. SVM showed higher accuracy (77%) than the other algorithms. The fifth experiment was aimed at differentiating between factory workers with and without exposure to DEE. In this case, KNN classified the dataset optimally and was able to distinguish between the two groups with an accuracy of 90%, while RF and DT displayed low accuracies. The sixth experiment was performed to classify the individuals by occupation, obesity, and smoking habits. When classifying by occupation, all the algorithms showed low accuracy. KNN showed good accuracy of 80.3% in the classification based on obesity. However, all algorithms were also inadequate in classifying the same dataset based on smoking and displayed a mean accuracy of 61.5%.

**Figure 13:**
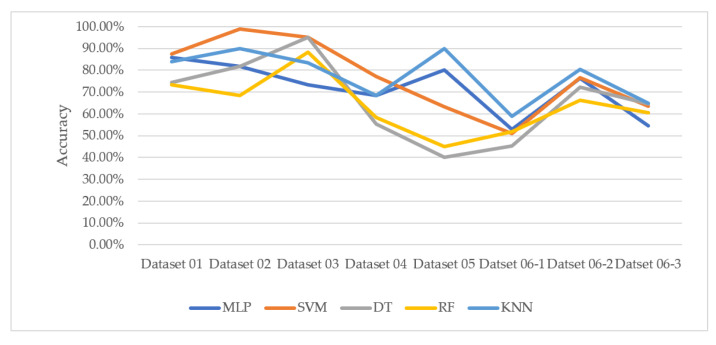
Accuracies for the tested classification algorithms.

Figure 13 summarizes the accuracies of each machine learning classification algorithm on all datasets used in the experiments. Datasets 01–04 concerned specific diseases, such as lung cancer, CVD, and COPD, while datasets 05–06 compared the data between two different occupational groups. The machine learning model that showed the highest performance varied across datasets. Although SVM showed the highest performance on Datasets 01–04 and KNN showed the highest performance on Datasets 05–06, it is not reasonable to conclude that SVM or KNN is the best method for microarray data because only some of the various microarray data were tested in this study. However, it was obviously observed that the two tree-based methods, DT and RF, showed similar trends in results and the remaining three methods, MLP, SVM, and KNN, showed similar trends. Both DT and RF are implemented in a tree structure, so it seems that they show similar trends on microarray data. DT and RF generally showed worse performance than other methods except for the result of Dataset 03.

Furthermore, the heatmaps for Datasets 02 and 03, which were classified by SVM with substantially high performances, displayed clearer boundaries for *C* values than the heatmaps for other datasets where the performance of SVM was poorer.

**Figure 14:**
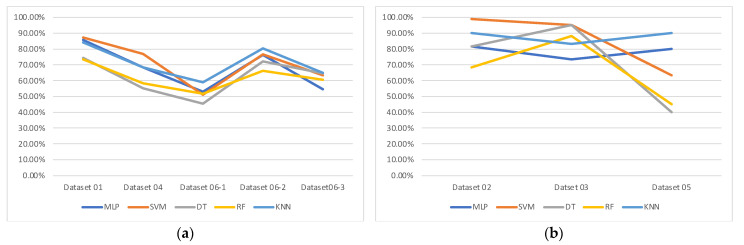
Results of the tested classification algorithms reorganized into miRNA and mRNA data; (**a**) accuracies for miRNA datasets; (**b**) accuracies for mRNA datasets.

Among the dataset used, Datasets 01, 04, and 06 are the miRNA datasets and others are the mRNA datasets. Figure 14 shows the accuracies of each machine learning classification algorithm on mRNA datasets and miRNA datasets separately. The difference in the performance of each model according to the two cases (miRNA and mRNA) was not clear. However, it can be observed that the performances of the tree-based models (RF, DT) were consistently worse than those of the distance-based model (MLP, SVM, KNN) with miRNA datasets. On the contrary, with mRNA datasets, the performances of the tree-based model were sometimes better or worse.

## 5. Conclusions

In this paper, microarray data with various traits were classified into two groups using various representative machine learning methods, MLP, SVM, DT, RF, and KNN. In the experiments, diverse classification criteria were applied, such as classification between two similar diseases, classification between people with and without diseases, and classification between two occupational groups. The accuracies by the five machine learning methods with these various datasets were compared. The results suggest that the best-performing machine learning model varies across datasets. However, it was observed that the tree-based methods, DT and RF, showed similar trends in results and the remaining methods, MLP, SVM, and KNN, showed similar trends. DT and RF generally showed worse performance than other methods except for one dataset.

Although only microarray data were dealt with in this paper, the methodology of this study is similarly applicable to RNA-seq data, which is known to be more sensitive in detecting differential expression and offers increased dynamic range. So, further study using the latest RNA-seq with the methods in this paper is needed. It will be interesting to verify whether similar results are derived in experiments using RNA-seq data as experiments on microarray data.

## Figures and Tables

**Table 1:** Six datasets used in our experiments.

No.	Dataset	Classification	Variables	Samples
01	MicroRNA profiling of chronic lung disease for the Lung Genomics Research Consortium	COPD vs. ILD	438	319
02	The site and nature of terminal bronchiolar destruction in chronic obstructive pulmonary disease (COPD)	COPD vs. control	19,718	117
03	Genome-wide screening of transcriptional modulation in non-smoking female lung cancer in Taiwan	Lung cancer vs. control	54,675	120
04	Differences in monocyte miRNA profiles between patients with coronary artery disease and healthy controls	CVD vs. control	461	105
05	Transcriptomic changes in the nasal epithelium associated with diesel engine exhaust exposure	Diesel vs. control	19,718	79
06	Expression of viral and human microRNAs in blood in the Beijing Truck Driver Air Pollution Study	Truck driver vs. office worker Obese vs. non-obese Smoker vs. non-smokers	734	252

**Table 2:** Example of miRNA dataset.

bkv-miR-B1-3p	3.024651	3.111211	3.32755	…
ebv-miR-BART10	3.657308	3.65909	3.785608	…
ebv-miR-BART12	7.352826	7.256859	6.621343	…
…	…	…	…	…
kshv-miR-K12-3	3.809819	4.034557	3.718077	…

**Table 3:** Parameters of classification algorithms.

Algorithm	Parameter	Explanation	Parameter Value
MLP	Hidden_layer_sizes	Size of the hidden layer	10, 10
	Activation	Activation function used in multilayer neural network	ReLU
	Solver	Function used for weight optimization	Adam
	Learning_rate	Controls the degree of weight update	0.01
	Max_iter	Maximum number of iterations	500
SVM	*C*	Controls tradeoff between smooth decision boundary and classifying training points correctly	0.001, 0.01, 0.1, 1, 10, 100
	γ	Defines how far the influence of a single training point reaches	0.001, 0.01, 0.1, 1, 10, 100
DT	Max_depth	Sets the maximum depth of tree	3
RF	N_estimators	Sets the number of decision trees.	100
KNN	N_neighbors	Number of neighbors to search	1–58

**Table 4:** Classification results for Dataset 01 (COPD/ILD).

	MLP	SVM	DT	RF	KNN
Accuracy	85.70%	87.40%	74.50%	73.30%	84.00%

**Table 5:** Classification results for Dataset 02 (COPD/control).

	MLP	SVM	DT	RF	KNN
Accuracy	81.70%	99.00%	81.70%	68.30%	90.00%

**Table 6:** Classification results for Dataset 03 (lung cancer/control).

	MLP	SVM	DT	RF	KNN
Accuracy	73.30%	95.00%	95.00%	88.30%	83.30%

**Table 7:** Classification results for Dataset 04 (CVD/control).

	MLP	SVM	DT	RF	KNN
Accuracy	68.30%	77.00%	55.30%	58.30%	68.30%

**Table 8:** Classification results for Dataset 05 (diesel factory worker/control).

	MLP	SVM	DT	RF	KNN
Accuracy	80.00%	63.30%	40.00%	45.00%	90.00%

**Table 9:** Classification results for Dataset 06 (truck driver/office worker).

	MLP	SVM	DT	RF	KNN
Accuracy	53.00%	51.00%	45.30%	51.70%	59.00%

**Table 10:** Classification results for Dataset 06 (Obesity/non-Obesity).

	MLP	SVM	DT	RF	KNN
Accuracy	76.30%	76.50%	72.30%	66.30%	80.30%

**Table 11:** Classification results for Dataset 06 (smoker/non-smoker).

	MLP	SVM	DT	RF	KNN
Accuracy	54.50%	63.50%	64.50%	60.50%	65.00%

## Data Availability

The data presented in this study are openly available in reference number [24,25,26,27,28,29].

## Supplementary Materials

The following supporting information can be downloaded at: https://www.mdpi.com/article/10.3390/genes13030494/s1, File S1: codes.

## Appendix A. The Effect of Normalization and Standardization

The effect of normalization and standardization for all the experiments performed in this study was compared through experiments. Table A1 compares the accuracy of classification algorithms according to normalization and standardization. The result averaged the accuracy of all experiments performed in this study. The higher accuracy between standardization and normalization was expressed in bold. The better feature scaling method was different depending on the machine learning model. According to the table, normalization showed better performance for SVM, DT, and KNN, and standardization showed better performance for MLP and RF. Normalization was better on average over all machine learning algorithms with the microarray data used in this study.

**Table A1:** The average of the accuracy obtained through eight experiments after feature scaling using normalization and standardization.

	MLP	SVM	DT	RF	KNN	AVG
Normalization	70.40%	**78.49%**	**69.80%**	66.67%	**75.70%**	**72.21%**
Standardization	**73.11%**	66.38%	69.49%	**71.31%**	65.77%	69.21%
